# A New Strategy to Integrate Heath–Carter Somatotype Assessment with Bioelectrical Impedance Analysis in Elite Soccer Players

**DOI:** 10.3390/sports8110142

**Published:** 2020-10-27

**Authors:** Francesco Campa, Tindaro Bongiovanni, Catarina N. Matias, Federico Genovesi, Athos Trecroci, Alessio Rossi, F. Marcello Iaia, Giampietro Alberti, Giulio Pasta, Stefania Toselli

**Affiliations:** 1 Department for Life Quality Studies, University of Bologna, 47921 Rimini, Italy; francesco.campa3@unibo.it; 2 Department of Health, Performance and Recovery, Parma Calcio 1913, 40121 Parma, Italy; 3 Department of Biomedical Sciences for Health, Università degli Studi di Milano, 20129 Milano, Italy; Athos.Trecroci@unimi.it (A.T.); marcello.iaia@unimi.it (F.M.I.); giampietro.alberti@unimi.it (G.A.); 4 Faculdade de Educação Física e Desporto, Universidade Lusófona, 1749-024 Lisboa, Portugal; cmatias@fmh.ulisboa.pt; 5 CIPER—Interdisciplinary Center for the Study of Human Performance, Faculty Human Kinetics, University of Lisbon, 1495-751 Lisboa, Portugal; 6 Bioperformance & Nutrition Research Unit, Ingrediente Métrico S.A., 2740-262 Lisbon, Portugal; 7 Medical Department Manchester City Football Club, Manchester 03101, UK; fede.genovesi@libero.it; 8 Department of Computer Science, University of Pisa, 56121 Pisa, Italy; alessio.rossi2@gmail.com; 9 Medical Department Parma Calcio 1913, 40121 Parma, Italy; ghitopasta@hotmail.com; 10 Department of Biomedical and Neuromotor Sciences, University of Bologna, 40126 Bologna, Italy; Stefania.toselli@unibo.it

**Keywords:** anthropometry, BIA, body composition, morphology, predictive equation

## Abstract

Easy-to-apply and quick methods for evaluate body composition are often preferred when assessing soccer teams. This study aimed to develop new equations for the somatotype quantification that would reduce the anthropometric measurements required by the Heath and Carter method, integrating the somatotype assessment to the bioelectrical impedance analysis (BIA). One hundred and seventy-six male elite soccer players (age 26.9 ± 4.5 years), registered in the Italian first division (Serie A), underwent anthropometric measurements and BIA. Endomorphy, mesomorphy, and ectomorphy were obtained according to the Heath and Carter method, while fat mass (FM) and fat free mass (FFM) estimated using a BIA-derived equation specific for athletes. The participants were randomly split into development (*n* = 117) and validation groups (*n* = 59, 1/3 of sample). The developed models including resistance^2^/stature, FM%, FFM, contracted arm and calf circumference, triceps, and supraspinal skinfolds had high predictive ability for endomorphy (R^2^ = 0.83, Standard Error of Estimate (SEE) = 0.16) mesomorphy (R^2^ = 0.80, SEE = 0.36), and ectomorphy (endomorphy (R^2^ = 0.87, SEE = 0.22). Cross validation revealed R^2^ of 0.80, 0.84, 0.87 for endomorphy, mesomorphy, and ectomorphy, respectively. The proposed strategy allows the integration of somatotype assessment to BIA in soccer players, reducing the number of instruments and measurements required by the Heath and Carter approach.

## 1. Introduction

Body composition evaluation is among the most common assessments used on soccer players, given its relationship with physical performance [1]. It has been stated that body composition variables are related to the ability to express power and strength, as well as to improve movement patterns in soccer players [2,3]; the interest in body composition also concerns aspects related to the athlete’s monitoring during the follow-up after a muscle injury [4].

Body composition can be interpreted and measured according to five organizational levels [5]. An exhaustive assessment includes measurement of variables belonging to different levels, such as fat mass (FM), fat-free mass (FFM), total body water (TBW), bone mineral content, intra (ICW), and extra cellular (ECW) fluids, and different anthropometric measurements and index [6]. In particular, FM and FFM belong to the molecular level, while the anthropometric and morphological characteristics are included in the fifth interpretative level (whole-body level). In this regard, endomorphy, mesomorphy, and ectomorphy represent the three morphological components of the somatotype and their different combinations allows for the classification of the athlete’s body shape in one of thirteen different categories [7].

Anthropometric features and somatotype vary according to the sport practiced and the position played [8,9]. Generally, volleyball and soccer players present an ectomorphic mesomorph somatotype, while rugby players are characterized by a dominance of the endomorphic component, especially those engaged in the defensive roles [8]. On the other hand, long distance runners show a balanced ectomorphic somatotype, being that ectomorphy is representative of linearity and correlated with better endurance performance [10]. The interest towards the evaluation of morphological characteristics in addition to body composition parameters belonging to the molecular level, such as FM and FFM, is recently increasing. Particularly, this occurs in high-level sports in order to obtain an optimal body composition profile and therefore predisposing the athletes to achieving high performance [11,12].

The measurement of a wide range of body composition variables requires different measuring instruments and specific expertise. Unfortunately, the use of reference methods for body composition assessment such as hydrostatic weighing, dilution techniques, and dual X-ray absorptiometry is not always possible due to the high cost and experience required. Bioelectrical impedance analysis (BIA) allows for the estimation of elements such as FM, FFM, and the measurement of raw parameters such as phase angle, which represents the ICW/ECW ratio [13,14]. Conducting BIA is easy, inexpensive, and above all a quick to use, it is often used in large-scale evaluations. On the contrary, the somatotype assessment requires longer procedures and three different instruments as well as specific manual skills [15]. In fact, according to the approach proposed by Heath and Carter, the somatotype can be calculated by measuring two circumferences, skinfold thickness in four points, and two breadths as well as weight and height, for a total of ten body dimensions [7]. Therefore, the evaluation of the somatotype according to the Heath and Carter method requires a qualified anthropometrist and the dependence on the measurement instruments, including possible technical errors that militate against the accuracy of the evaluations. For this reason, BIA is often used in the body composition assessment of athletes, especially soccer players [16,17,18]. Recently, it has been demonstrated that bioelectric properties are able to discriminate the different somatotype categories in athletes, given the predictive role of their raw measurements with muscle mass and body fluids distribution, which vary according to the absolute FM and FFM content [8,19,20].

The development of a new strategy capable of reducing the number of anthropometric measurements required by the Heath and Carter method would enable the measurements of different body composition variables using a simpler and faster procedure. Reducing the number of instruments used and the measurements collected for the evaluation of the somatotype would permit the integration of the analysis of morphological characteristics to BIA, thus allowing the analysis of a large number of variables belonging to different levels of body composition in a practical way and in short amount of time. Therefore, this study aimed to develop and validate new equations by integrating anthropometric and bioimpedance measurements in order to estimate the three somatotype components in elite soccer players.

## 2. Materials and Methods

### 2.1. Participants

One hundred and seventy-six male soccer players (age 26.9 ± 4.5 years), registered in a professional Italian soccer team participating in the first division (Serie A) were selected to participate in the study (*n* = 8 goalkeepers, *n* = 50 defenders, *n* = 62 midfielders, and *n* = 56 forwards). The players voluntarily decided to participate and provided informed consent after a detailed description of the study procedures. The project was conducted according to the Declaration of Helsinki and was approved by the Bioethics Committee of the University of Bologna (Approval Code: 25027).

### 2.2. Procedures

All anthropometric measurements were profiled by a 1 Level ISAK accredited anthropometrist (T.B.) following the International Society Advancement Kinanthropometry guidelines [21]. The technical error of measurement was within 5% agreement for skinfolds and within 1% for breadths and girths. Height was recorded to the nearest 0.1 cm with a standing stadiometer (Seca 217, Basel, Switzerland), and body weight was measured to the nearest 0.1 Kg with a high-precision mechanical scale (Seca 877, Basel, Switzerland). Body mass index (BMI) was calculated as the ratio of body mass to height squared (kg/m^2^). Girths were taken to the nearest 0.1 cm using a tape measure (Lufkin executive thinline, W606ME) and breadths using a sliding caliper (GMP, Zürich, Switzerland), while skinfold thicknesses were measured to the nearest 0.1 mm using a skinfold caliper (Holtain Ltd, Crymych, UK). Somatotype components were calculated according to the Heath–Carter method [7]. The raw impedance parameters, resistance (R) and reactance (Xc) were obtained with a bioimpedance analyzer (BIA 101 Anniversary; Akern Srl, Florence, Italy) at a frequency of 50 kHz, according to the standard procedures [22,23]; bioimpedance parameters were measured in the morning (9:00 a.m.) by using the standard positions of outer and inner electrodes on the right hand and foot and the analyzer calibration was checked before each measurement. The athletes were in a supine position five minutes before the test, which was performed in a thermoneutral environment of 25 °C. Phase angle (PhA) was calculated as follows:

PhA = Xc/R × 180°/π.
FFM, FM, and FM% using a specific equation for athletes as follow [24]:
FFM = −2.261 + 0.327 × stature2/R + 0.525 × body weight + 5.462 × 1;
FM = Body weight − FFM;
FM% = FM/body weight × 100.

### 2.3. Statistical Analysis

Descriptive statistics was performed to characterize the sample. Normality of the investigated variables was assessed using the Kolmogorov–Smirnov test. Stratified random assignment was used to assign participants to either a development group (*n* = 117) or a cross validation group (*n* = 59). Stepwise regression analysis was used to evaluate the ability of variables (age, R, Xc, PhA, FFM, FM, FM%, FFM/stature, stature, weight, BMI, skinfold thickness, circumferences, stature^2^/resistance, and stature^2^/reactance) to predict endomorphy, mesomorphy, and ectomorphy in the development group. During model development, normality of residuals and homogeneity of variance were tested. The criterion for inclusion of a predictor was significance at *p* ≤ 0.05, removal criteria were set at *p* ≤ 0.10. If more than one variable remained in the model, a variance inflation factor (VIF) for each independent variable was calculated, and values below five were considered as not having multicollinearity. To cross-validate the developed models, the resulting equations were applied to the cross-validation group according to the statistics method described elsewhere [25]. To assess the accuracy of the new predictive models, validation parameters included the analysis of the coefficient of determination and the pure error. The pure error was assessed using the following equation (∑(Y¨–Y)2/n1/2, where Y¨ is the predicted variable, Y is the observed variable and *n* is the number of participants [26]. Additionally, the concordance correlation coefficient (CCC) using the Lin approach [27] was performed. The CCC contains a measurement of precision and accuracy (ρc = ρ Cb): where ρ is the Pearson correlation coefficient, which measures how far each observation deviates from the line of best-fit and is a measure of precision, and Cb is a bias correction factor that measures how far the best-fit line deviates from the 45° line through the origin, and is a measure of accuracy. Finally, agreement between developed models and the reference procedure was assessed using the Bland–Altman method [28], including the analysis of the correlation between the mean and the difference of the methods and an estimate of the limits of agreement. Data were analyzed with IBM SPSS Statistics, version 24.0 (IBM Corp., Armonk, NY, USA).

## 3. Results

Table 1 shown the general characteristics of the soccer players divided into development and validation groups. Considering the whole sample, the 96.2% of the soccer players were of Caucasian ethnicity.

No significant interaction with player role and ethnicity for any of the main independent predictors. Table 2 shows the final developed prediction equations for estimating endomorphy, mesomorphy, and ectomorphy. FM%, S^2^/R, triceps and supraspinal skinfolds, and stature explained 83% of the variance in Heath and Carter measured endomorphy. Contracted arm and calf circumferences, FFM, and stature explained 80% of the variance in mesomorphy, while FFM/stature explained 87% of the variance in ectomorphy.

**Figure 1:**
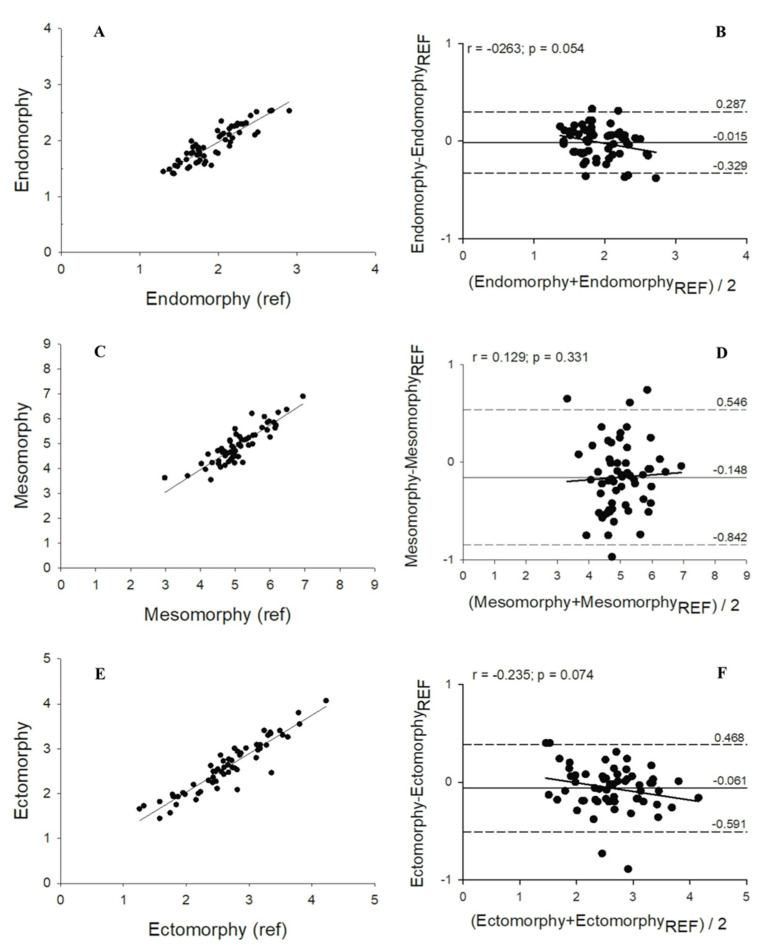
On the left side the scatterplots with the relationship between the predicted and the reference somatotype components (**A**) = endomorphy, (**C**) = mesomorphy, (**E**) = ectomorphy. On the right side the results of Bland–Altman analyses (**B**) = endomorphy, (**D**) = mesomorphy, (**F**) = ectomorphy.

Regarding the regression analysis, the methods were highly correlated (R^2^ ≥ 0.80; *p* < 0.001). The predictive models developed in this study for the three somatotypes explained 80%, 84%, and 87% of the variability observed in the values of the reference methods for endomorph, mesomorph, and ectomorph somatotypes, respectively (Figure 1). The precision and accuracy of the methods was higher than 0.89 and 0.99, respectively, with a CCC between the new method and the reference procedure superior to 0.89 (Table 3). From the agreement analysis, we observed no trend between the mean and the differences of the methods for any of the somatotypes, with limits of agreement considered acceptable.

## 4. Discussion

The objective of this study was to propose a new strategy to integrate BIA and anthropometric measurements to estimate the three somatotype components in elite soccer players. The new proposed models allow for the assessment of the endomorphy, mesomorphy, and ectomorphy components by reducing the number of measurements and instruments required by the traditional Heath and Carter method. The equations developed in this study include anthropometric, bioelectrical, and BIA-derived body composition parameters. This allows for the evaluation of body composition variables belonging to the molecular (FM, FFM, and TBW), cellular (ICW and ECW), and whole-body (somatotype) levels, in a reduced time and with fewer operator dependent errors. 

A cross validation was performed, and a very strong correlation was observed between the developed equations and the reference method (R^2^ ≥ 0.80). Additionally, precision and accuracy between the new predictive equation and the reference procedure were assessed with concordance correlation coefficient analysis. A moderate strength of agreement [29] between the methods was observed in estimating mesomorph and ectomorph somatotypes (CCC = 0.90 and 0.93, respectively), while for the endomorph somatotype, a less strong agreement was observed between the methods (CCC = 0.89). The magnitude of the differences between the new predictive model and the reference method was examined according to the Bland–Altman method [28] where no significant trend was observed, and the 95% confidence intervals were acceptable. However, while the results showed high correlations, estimated mesomorph had relatively wider variability compared with other two components of somatotype. This may be due to the missing breadth measurements in the predictive model, which represents both a limitation in accuracy but also an advantage in its ease of application and time efficiency.

In this study, the measured bioelectrical parameters and somatotypes are in line with previously reported research on elite soccer players. In fact, a PhA of 7.9° was measured in this study and by Levi et al. [17] and Mascherini, et al. [18] where soccer players presented values of 7.7°and 8.0°, respectively. PhA represents the ICW/ECW ratio [14,30] and is considered a cellular health biomarker as well as representative of the changes in fluids that occur as a result of muscles injuries. In this regard, Nescolarde et al. [4] proposed PhA as a parameter capable of identifying the restoration of cell membranes that follow the return-to-play after a muscle lesion. The somatotype measured in our athletes was ectomorphic-mesomorph, as also suggested by other recent studies on high-level soccer players [8,9]. This indicates that the soccer player of the new millennium is an athlete who has both a high muscle mass and important physical dimensions. In addition, some studies have reported that roles such as external defender and midfielders [19], which have to cover greater distances during the match, may have a greater ectomorphic component than the mesomorphic one. In fact, better endurance characteristics can be found in ectomorphic athletes, such as runners [10]. 

It has been shown how the somatotype assessment can provide meaningful information in sports where morphology can impact the biomechanical movement and resulting performance [31,32]. Quantification of somatotype in elite athletes plays an important role in monitoring the body shape changes which occur in response to competitive and training periods. In fact, previous studies have already shown how through the anthropometric profile assessment it is possible to determine the player’s suitability for high-level competitions [33,34]. Furthermore, while several studies have shown the relationship between strength and power with mesomorphy [35,36], recently Ryan et al. [37] found that a mosomorph–ectomorph somatotype are associated with higher expression of maximum strength.

It is essential to highlight the strength of this study which is represented by the large sample size of elite soccer players. This allowed for the creation of two different groups, one for development and one for equation validations. This represents an important point as the accuracy of the predictive models was ensured on a different sample other than the one on which they were developed.

However, some limitations should be addressed. First of all, these equations may not be applicable to athletes practicing sports other than soccer or to athletes of a different gender. Secondly, the new models may lose accuracy when applied to BIA devices working at a different frequency than that used in this study [38]. Lastly, the new BIA-derived equations are estimative models that always present an inherent level of error in relation to the components of the somatotype; given that the bioelectrical properties are directly informative of body fluids and cell density [23]. Furthermore, BIA may be much more expensive than the simple use of anthropometric instruments, especially when making measurements on large samples.

## 5. Conclusions

Quantification of somatotype components can provide important insight into body composition research. The traditional method proposed by Heath and Carter requires the use of different instruments and long measurement times often unsuitable for field evaluations. In this study, three new equations for estimating somatotype components in soccer players are provided. The new strategy proposed in this study allows for a minimal number of measurements and instruments for assessing somatotype, integrating anthropometry and BIA for an exhaustive evaluation of the body composition in soccer players.

## Figures and Tables

**Table 1:** Descriptive characteristics of the development and cross-validation groups.

Variable	Development Group (*n* = 117)	Cross-Validation Group (*n* = 59)
	Mean ± Standard Deviation	Mean ± Standard Deviation
Age (years)	27.4 ± 4.3	28.0 ± 5.0
Weight (kg)	79.5 ± 6.1	78.9 ± 6.5
Stature (cm)	183.8 ± 0.5	182.9 ± 0.5
Body mass index (kg/m^2^)	23.5 ± 1.2	23.6 ± 1.3
Resistance (ohm)	464.3 ± 37.1	456.8 ± 33.3
Reactance (ohm)	63.8 ± 5.7	64.6 ± 6.6
Phase angle (degree)	7.9 ± 0.5	8.1 ± 0.9
Fat-free mass (kg) Fat mass (kg) Fat mass (%)	68.8 ± 5.4	68.7 ± 5.7
	10.6 ± 1.7	10.2 ± 1.9
	13.3 ± 1.9	12.9 ± 1.9
Triceps skinfold (mm)	6.1 ± 1.7	5.6 ± 1.2
Subscapular skinfold (mm)	9.5 ± 1.7	9.6 ± 1.7
Supraspinal skinfold (mm)	6.7 ± 1.8	6.4 ± 1.7
Medial calf skinfold (mm)	5.4 ± 1.4	5.0 ± 0.9
Contracted arm circumference (cm)	33.1 ± 1.7	33.3 ± 1.5
Calf circumference (cm)	37.9 ± 1.7	38.2 ± 3.4
Humerus width (cm)	7.2 ± 0.4	7.2 ± 0.3
Femur width (cm)	10.2 ± 0.5	10.3 ± 0.5
Endomorphy	2.0 ± 0.4	1.9 ± 0.4
Mesomorphy	4.9 ± 0.8	5.2 ± 0.8
Ectomorphy	2.7 ± 0.6	2.7 ± 0.6

**Table 2:** Prediction models for endomorphy, mesomorphy, and ectomorphy based on anthropometrics and bioimpedance-derived variables.

Variable	Predictors	R	R^2^	SEE	VIF	Prediction Equation
Endomorphy	FM% S^2^/R Triceps skinfold Supraspinal skinfold Stature	0.91	0.83	0.16	2.12 3.45 1.06 1.15 2.29	y = 4.292 + 0.050 × FM% + 0.012 × S^2^/R + 0.092 × triceps skinfold + 0.139 × supraspinal skinfold − 0.029 × stature
Mesomorphy	CAC CC FFM Stature	0.89	0.80	0.36	1.58 2.11 4.80 2.54	y = 10.351 + 0.212 × CAC + 0.187 × CC + 0.048 × FFM − 0.125 × stature
Ectomorphy	FFM/S Stature	0.94	0.87	0.22	1.29 1.29	y = −7.945 − 25.021 × FFM/S + 0.109 × Stature

Abbreviations: R = multiple correlation coefficient; R^2^ = multiple coefficient of determination; SEE = standard error of estimate; VIF = variation inflation factor; FM% = percentage of fat mass; S^2^/R = stature^2^/resistance; CAC = contracted arm circumference; CC = calf circumference; FFM = fat-free mass; FFM/S = fat-free mass/stature.

**Table 3:** Cross-validation of the somatotype predictive models and the reference procedure.

Variable	Regression Analysis		CCC Analysis			Agreement Analysis		
	R^2^	PE	CCC	ρ	C_b_	Bias	95% LoA	Trend
Cross-Validation								
Endomorph	0.80	0.162	0.89	0.8957	0.9917	−0.0149	−0.329; 0.287	r = −0.263 (*p* = 0.054)
Mesomorph	0.84	0.338	0.90	0.9173	0.9940	−0.1479	−0.842; 0.546	r = −0.129 (*p* = 0.331)
Ectomorph	0.87	0.229	0.93	0.9346	0.9973	−0.0613	−0.591; 0.468	r = −0.235 (*p* = 0.074)

Abbreviations: R^2^, coefficient of determination; PE, pure error; CCC, concordance correlation coefficient; ρ, precision; C_b_, accuracy; LoA, limits of agreement.
